# The dynamic shifts of IL-10-producing Th17 and IL-17-producing Treg in health and disease: a crosstalk between ancient "Yin-Yang" theory and modern immunology

**DOI:** 10.1186/s12964-024-01505-0

**Published:** 2024-02-06

**Authors:** Huantian Cui, Ning Wang, Hanzhou Li, Yuhong Bian, Weibo Wen, Xiangying Kong, Fudi Wang

**Affiliations:** 1 First School of Clinical Medicine, Yunnan University of Chinese Medicine, Kunming, 650500 China; 2 College of Integrative Chinese and Western Medicine, Tianjin University of Traditional Chinese Medicine, Tianjin, 301617 China; 3 Institute of Chinese Materia Medica, China Academy of Chinese Medical Sciences, Beijing, 100700 China; 4 The First Affiliated Hospital, Institute of Translational Medicine, The Second Affiliated Hospital, School of Public Health, Cancer Center, State Key Laboratory of Experimental Hematology, Zhejiang University School of Medicine, Hangzhou, 310058 China

**Keywords:** IL-10-producing Th17, IL-17-producing Treg, Immune system, Disease progression, Yin-Yang theory

## Abstract

The changes in T regulatory cell (Treg) and T helper cell (Th) 17 ratios holds paramount importance in ensuring internal homeostasis and disease progression. Recently, novel subsets of Treg and Th17, namely IL-17-producing Treg and IL-10-producing Th17 have been identified. IL-17-producing Treg and IL-10-producing Th17 are widely considered as the intermediates during Treg/Th17 transformation. These “bi-functional” cells exhibit plasticity and have been demonstrated with important roles in multiple physiological functions and disease processes. Yin and Yang represent opposing aspects of phenomena according to the ancient Chinese philosophy “Yin-Yang” theory. Furthermore, Yin can transform into Yang, and vice versa, under specific conditions. This theory has been widely used to describe the contrasting functions of immune cells and molecules. Therefore, immune-activating populations (Th17, M1 macrophage, etc.) and immune overreaction (inflammation, autoimmunity) can be considered Yang, while immunosuppressive populations (Treg, M2 macrophage, etc.) and immunosuppression (tumor, immunodeficiency) can be considered Yin. However, another important connotation of “Yin-Yang” theory, the conversion between Yin and Yang, has been rarely documented in immune studies. The discovery of IL-17-producing Treg and IL-10-producing Th17 enriches the meaning of “Yin-Yang” theory and further promotes the relationship between ancient “Yin-Yang” theory and modern immunology. Besides, illustrating the functions of IL-17-producing Treg and IL-10-producing Th17 and mechanisms governing their differentiation provides valuable insights into the mechanisms underlying the dynamically changing statement of immune statement in health and diseases.

## Introduction

As a crucial component of the adaptive immune system, T lymphocytes play pivotal roles in various physiological processes, including pathogen defense and the maintenance of internal homeostasis. Among these processes, the dynamic equilibrium between T regulatory cell (Treg) and T helper cell (Th) 17 holds paramount importance in ensuring internal homeostasis [1]. Both Treg and Th17 subsets are CD4^+^ T cells, with distinct characteristics and functions. Tregs express CD25, forkhead box P3 (Foxp3), and interleukin (IL)-10 and differentiate from CD4^+^ T cells upon transforming growth factor (TGF)-β1 stimulation [2]. Tregs produce immunosuppressive factors such as IL-10 and TGF-β1, which are involved in dampening inflammatory responses, promoting tissue repair, and maintaining immune tolerance [3]. Conversely, Th17s are characterized by the expression of transcription factor retinoic acid-related orphan nuclear receptor γt (RORγT) and the production of interleukin IL-17. Their differentiation from native CD4^+^ T cells is induced by TGF-β1 and IL-6 stimulation [4]. Th17 cells secrete several cytokines, including IL-17, IL-21, and IL-23, which contribute to pro-inflammatory responses [5].

Imbalances in the Treg/Th17 ratio are implicated in the progression of various inflammatory responses [6], autoimmune diseases [6], and tumors [7]. In inflammatory and autoimmune diseases, an increase in Th17 differentiation and inadequate Treg differentiation leads to excessive release of pro-inflammatory factors, resulting in overactive immune responses [3]. In the context of tumor development, a significant portion of CD4^+^ T cells differentiates into Tregs, enabling tumor cell immune evasion [8]. Concurrently, Th17-produced inflammatory factors in tumor tissues can influence the tumor microenvironment and impact tumor progression through various mechanisms [9].

Notably, recent studies have identified novel subsets of Tregs and Th17 cells, namely IL-17-producing Treg [10] and IL-10-producing Th17 [11]. These “bi-functional” cells exhibit plasticity and play significant roles in multiple physiological functions and disease processes.

### IL-17-producing Treg

IL-17-producing Tregs were initially reported by Kui Shin Voo et al. from the University of Texas in 2009 [10]. These cells were first identified in human peripheral blood and lymphoid tissue, accounting for 0.32% and 2.4% of CD4^+^ T cells in human peripheral blood and tonsils, respectively. Functional assessments of these cells revealed that both IL-17-producing Tregs and conventional Tregs (Foxp3^+^ IL-17^−^) were capable of significantly inhibiting T cell activation induced by anti-CD3 and anti-CD28. In contrast, conventional Th17 (Foxp3^−^IL-17^+^ T cells) or Foxp3^−^IL-17^−^ T cells did not exhibit this inhibitory function, highlighting that IL-17-producing Tregs share immunosuppressive properties with Tregs [10, 12].

### The characteristics and physiological function of IL-17-producing Treg

**Fig. 1:**
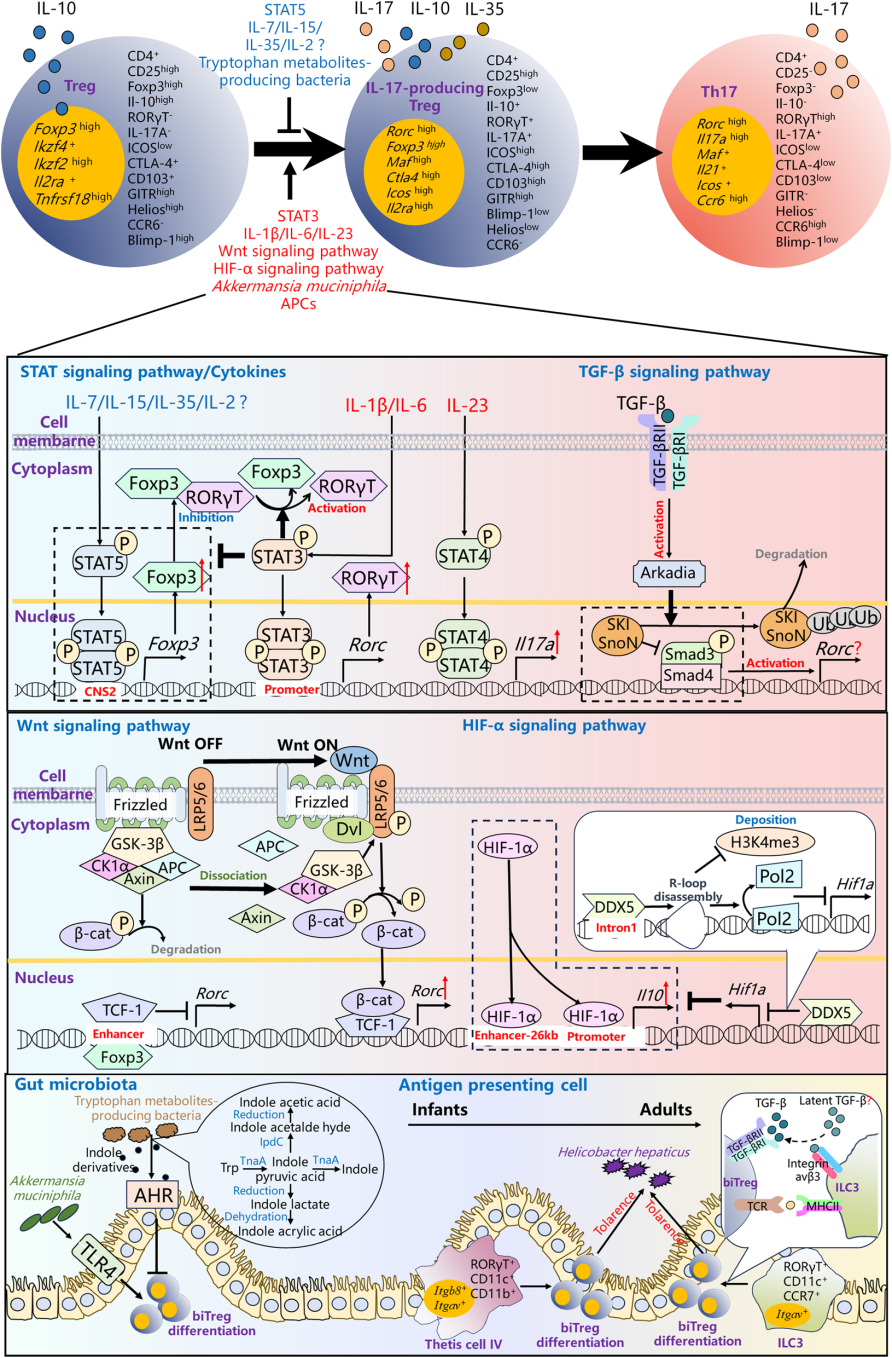
Regulation of IL-17-producing Treg differentiation. Tregs could convert into IL-17-producing Tregs, releasing both IL-10 and IL-17 A. Subsequently, IL-17-producing Treg could further convert into Th17 cells. IL-1β, IL-6 and IL-23 could induce IL-17-producing Treg differentiation through activating STAT3, whereas IL-7, IL-15, IL-35 and IL-2 may inhibit IL-17-producing Treg differentiation due to their active roles on STAT5. Activation of Wnt signaling pathway contributed to IL-17-producing Treg differentiation through upregulating *Rorc* expression and HIF-1α enhanced the IL-10-producing capacity in IL-17-producing Tregs. *Akkermansia muciniphila* induced IL-17-producing Treg differentiation through the activation of TLR4, whereas tryptophan metabolites-producing bacteria inhibit IL-17-producing Treg differentiation through AhR. In infants, a newly discovered APC (Thetis cell IV) in gut induced IL-17-producing Treg differentiation and ILC3 induced IL-17-producing Treg differentiation in adults

IL-17-producing Tregs have been identified not only in peripheral blood and lymphoid tissue but also in various organs, including the intestine [13], spleen [14], lymph nodes [14], lung [15], liver [15], kidney [16], and other tissues. In contrast to conventional Tregs, IL-17-producing Tregs exhibit distinct phenotypic characteristics, characterized by high levels of RORγT, inducible co-stimulator (ICOS), cytotoxic T-lymphocyte-associated antigen 4 (CTLA-4), and CD103 expression. Notably, they show lower expression levels of Foxp3 and B-lymphocyte-inducing maturation protein 1 (Blimp-1), along with moderate levels of C-C chemokine receptor 6 (CCR6), CD25, and glucocorticoid induced TNF receptor (GITR) [14]. Additionally, these cells exhibit reduced expression of Th1-related markers, including interferon gamma (IFN-γ) and C-X-C motif chemokine receptor 3 (CXCR3) [17]. Consequently, IL-17-producing Tregs are often described as RORγT^+^ Tregs (Fig. 1). Currently, immunofluorescence, flow cytometry, and single-cell sequencing are commonly used to investigate IL-17-producing Treg in multiple tissues. The detailed information for investigating IL-17-producing Treg is shown in Table 1.

**Table 1:** Investigation of IL-17-producing Treg and IL-10-producing Th17

Cell type	Source	Detection index	Method	Reference
IL-17-producing Treg	Lymph node	RORγT^+^Foxp3^+^	Flow cytometry	[18]
	Ankle joints	RORγT^+^Foxp3^+^	Immunofluorescence	[18]
	Colon and spleen	CD4^+^TGF-β^+^RORγT^+^Foxp3^+^	Flow cytometry	[19]
	Lymph node	CD4^+^RORγT^+^Foxp3^+^	Flow cytometry	[20, 21]
	Colorectal cancer	CD4^+^Foxp3^+^IL-17A^+^	Flow cytometry	[22]
	T cell in vitro	CD4^+^CD25^high^Foxp3^+^IL-17A^+^	Flow cytometry	[22]
	Peripheral blood	CD4^+^Foxp3^+^IL-17A^+^	Flow cytometry	[10, 23]
	Mucosa	CD4^+^CD25^+^CD127^−^IL-17A^+^	Single-cell sequencing	[24]
IL-10-producing Th17	Small intestinal T cells in Fate^+^ mice	CD4^+^YFP^+^IL-10^GFP+^IL-17A^katushka+^	Flow cytometry	[25]
	T cells in vitro	CD4^+^IL-10^+^IL-17A^+^	Flow cytometry	[26–28]
	Peritoneal fluid	IL-10^+^IL-17A^+^	Flow cytometry	[29]

Functionally, IL-17-producing Tregs secrete a combination of cytokines, including IL-17, IL-10, and IL-35 [14]. Under physiological conditions, IL-17-producing Tregs play a crucial role in safeguarding organs from external stimuli and inflammatory injury, as evidenced by their anti-bacterial infection and inflammation repression functions (Fig. 1) [10]. Commensal bacteria, such as *Helicobacter*, have been shown to induce the differentiation of IL-17-producing Tregs, promoting immune tolerance and preventing excessive inflammation. Notably, the absence of IL-17-producing Tregs renders individuals more susceptible to microbially induced mucosal inflammation [30]. Comparison of the gene expression profiles of IL-17-producing Treg, Treg, and Th17 cells through transcriptomic analysis has revealed that IL-17-producing Tregs exhibit a gene expression pattern more akin to that of Treg cells. Furthermore, demethylation occurs in the CpG regions of Treg-related genes, such as *Foxp3, Ctla-4*, and *Helios*, within the IL-17-producing Treg genome. Importantly, IL-17-producing Tregs exert immune inhibitory effects when co-cultured with effector T cells [17].

A recent report in *Immunity* delves into the role of gut-derived IL-17-producing Tregs in regulating muscle regeneration [15]. Employing single-cell sequencing, researchers discovered that IL-17-producing Tregs are rarely present in normal skeletal muscle tissue, but their proportion significantly increases in muscle tissue following cardiotoxin (CTX)-induced muscle injury. This increase in IL-17-producing Tregs peaks three days after CTX injection, gradually declining and disappearing by day seven. Transplanting colon-derived CD4^+^ T cells into *Tcrb*^−/−^ mice revealed that CTX injection led to a notable increase in IL-17-producing Tregs in skeletal muscle, while transplantation of spleen-derived CD4^+^ T cells did not yield this effect, signifying the colon as the origin of IL-17-producing Tregs in injured skeletal muscle tissue. Additionally, IL-17-producing Tregs exhibit the ability to migrate to injured muscle tissue under the influence of C-C chemokine ligand 2 (CCL2) and major histocompatibility complex class II (MHC-II). Neutralizing antibodies targeting CCL2 and MHC-II effectively block the migration of intestinal IL-17-producing Tregs to skeletal muscle. During the early stages of muscle injury, IL-17 and other pro-inflammatory cytokines stimulate muscle stem cell differentiation, promoting muscle regeneration [15]. However, in later stages, these pro-inflammatory cytokines induce muscle fibrosis, hindering muscle regeneration [31, 32]. Therefore, the increased presence of IL-17-producing Tregs in the early stage of injury (Day 3) contributes to IL-17 production and facilitates muscle regeneration. Subsequently, their numbers gradually diminish and disappear by Day 7, serving to control IL-17 levels and promote muscle repair. The removal of IL-17-producing Tregs in mice significantly impairs muscle repair ability [15].

### Regulatory factors involved in IL-17-producing Treg differentiation

Presently, the consensus among researchers is that IL-17-producing Tregs represent an intermediate stage in the differentiation of Tregs into Th17 cells [33]. Notably, peripheral blood IL-17-producing Tregs can differentiate from thymic Tregs, and substantial differences in gene expression patterns exist between IL-17-producing Tregs in the gut and those in peripheral blood, implying that IL-17-producing Tregs can originate from multiple organs [19, 20]. It is essential to note, however, that one study contradicts this perspective by suggesting that IL-17-producing Tregs are not intermediates in the conversion of Tregs into Th17 cells [14]. These varying findings may stem from differences in the sources of IL-17-producing Tregs. Multiple regulatory factors have been identified as influencing the differentiation of IL-17-producing Tregs, including STAT signaling pathways, cytokines, TGF-β, autophagy, Wnt/β-catenin, hypoxia-inducible factor-1 alpha (HIF-1α), gut microbiota, costimulatory molecules, microRNA (miRNA), and others. These factors collectively contribute to the complex and finely tuned process of IL-17-producing Treg differentiation (Fig. 1).

### Opposite functions of STAT3 and STAT5 in regulating IL-17-producing Treg differentiation

Key transcription factors, Foxp3 and RORγT, dictate the differentiation of CD4^+^ T cells into Tregs or Th17s, respectively [34]. Foxp3 plays a pivotal role in the maturation, development, and execution of immunosuppressive functions by Tregs [35, 36]. It upregulates the expression of immunosuppressive factors such as IL-10 and TGF-β1, inhibits the transcriptional activity of RORγT, and represses the expression of the pro-inflammatory factor gene IL-17 [37–40]. Conversely, RORγT acts as a critical transcription factor in Th17 differentiation, driving the upregulation of IL-17 expression while diminishing Foxp3 transcription and suppressing IL-10 and TGF-β1 expression [41]. Changes in the expression levels of Foxp3 and RORγT serve as crucial indicators of cell differentiation towards IL-10 and IL-17 secretion.

Within the realm of regulating Foxp3 and RORγT, the STAT3 and STAT5 transcription factors play pivotal roles during IL-17-producing Treg differentiation. STAT3 and STAT5 are composed of an N-terminal interaction domain, a coiled helix domain, a DNA-binding domain, a ligand domain, an Src homology 2 (SH2) domain, and a C-terminus transcriptional activation domain [42]. Upon tyrosine kinase induction, STAT3 and STAT5 undergo phosphorylation, leading to the formation of phosphorylated STAT3 or STAT5 homodimers through the N-terminal interaction domain, subsequently translocating to the nucleus. STAT3 binds to the promoter region of the *Rorc* gene, promoting *Rorc* transcription and inducing IL-17 production [43]. Conversely, STAT5 binds to the conserved non-coding sequence 2 (CNS2) region of the *Foxp3* gene, enhancing *Foxp3* expression and facilitating IL-10 production [44]. Additionally, STAT3 can promote RORγT activation by facilitating the dissociation of RORγT from Foxp3 and relieving the inhibitory effect of Foxp3 on RORγT [45]. Maintaining a balance between STAT3 and STAT5 signaling is a crucial factor in Treg/IL-17-producing Treg differentiation (Fig. 1). Elevated STAT3 phosphorylation levels in IL-17-producing Tregs enable these cells to express RORγT and possess the ability to produce IL-17. Promoting STAT3 phosphorylation while inhibiting STAT5 phosphorylation can induce the differentiation of Tregs into IL-17-producing Tregs [20]. Mice with conditional STAT3 knockout (*Cd4*^cre^*Stat3*^fl/fl^) in CD4^+^ cells fail to produce IL-17-producing Tregs [20].

### Promotive roles of IL-2, IL-1β, IL-6, IL-21, IL-23 and suppressive roles of IL-7, IL-15, IL-35 in IL-17-producing Treg differentiation

The differentiation of IL-17-producing Tregs is subject to regulation by an array of cytokines. IL-2, IL-1β, IL-6, IL-21, and IL-23 have been identified as promoters of IL-17-producing Treg differentiation [46–51]. Among these cytokines, both IL-1β and IL-6 activate STAT3, leading to the induction of RORγT expression and the stimulation of T cell production of IL-17 A [12, 46, 52]. Additionally, IL-23 stabilizes IL-17 production through STAT4 [53]. IL-2 can induce CCR6^+^CD4^+^CD25^high^ T cells to differentiate into IL-17-producing Tregs, while the combination of IL-2 with IL-1β, IL-6, IL-21, or IL-23 can induce CCR6^−^CD4^+^CD25^high^ T cells to differentiate into IL-17-producing Tregs [10]. However, it is noteworthy that there is conflicting evidence regarding the impact of IL-2 on IL-17-producing Treg differentiation, possibly attributed to its role in activating STAT5 and inducing Foxp3 expression [54, 55]. The influence of IL-2 on IL-17-producing Treg differentiation remains an area of ongoing research. Furthermore, cytokines such as IL-7 [56], IL-15 [57], and IL-35 [58] induce STAT5 phosphorylation and upregulate Foxp3 expression in Treg cells, thereby maintaining Treg homeostasis, which may hinder IL-17-producing Treg differentiation (Fig. 1). Nevertheless, the potential effects of these cytokines on IL-17-producing Tregs warrant further investigation.

### Intricate mechanism of TGF-β in inducing IL-17-producing Treg differentiation

TGF-β plays a crucial role in the differentiation of both Treg and Th17 [59]. While the role of TGF-β in promoting Foxp3 expression and inducing Treg differentiation has been extensively studied [2], its role in Th17 differentiation remains enigmatic, despite the antagonistic functions of Foxp3 and RORγT [60]. Notably, IL-6 alone is insufficient to induce IL-17 secretion from T cells lacking suppressor of mothers against decapentaplegic (Smad)4, underscoring the crucial role of TGF-β in Th17 differentiation [61].

As previously mentioned, TGF-β is not only essential for inducing Th17 cells to produce IL-10 but also plays a pivotal role in IL-17-producing Treg differentiation. The combination of TGF-β and IL-1β can induce CCR6^−^CD4^+^CD25^high^ T cells to differentiate into IL-17-producing Tregs [10]. TGF-β promotes Foxp3 expression through the Arkadia-v-ski avian sarcoma viral oncogene homolog (Ski) / Ski-related novel protein N (SnoN) pathway, facilitating Treg differentiation. Ski/SnoN serve as inhibitors of Smads, recruiting transcriptional repressor Smad2/3/4 activity by binding to Smad2/3/4. Upon receptor binding, TGF-β activates the E3 ubiquitin ligase Arkadia, which ubiquitinates Ski/SnoN, leading to their degradation. This process derepresses Smad2/3/4 transcription and upregulates Foxp3 expression (Fig. 1) [62, 63]. Intriguingly, CD4^+^ T cells with specific *Arkadia* deletion (*Cd4*^cre^*Arkadia*^fl/fl^) display reduced Treg differentiation capacity. Remarkably, these mice exhibit a significant reduction in IL-17-producing Treg differentiation but no changes in Th17 cell differentiation compared to wild-type mice [61]. This observation suggests that Arkadia has no effect on RORγT expression. The mechanisms underlying Smad signaling pathways’ regulation of RORγT expression remain unclear. Moreover, variations in TGF-β sources, environmental factors such as cytokines and temperature, and intricate interactions between TGF-β and other signaling pathways may contribute to this phenomenon [61]. Future research is expected to unveil the mechanistic basis for TGF-β induction of IL-17-producing Treg differentiation.

### Autophagy: an essential factor in IL-17-producing Treg differentiation

Autophagy, a vital intracellular metabolic process, plays a crucial role in maintaining cellular function and homeostasis by facilitating the degradation and recycling of damaged proteins, organelles, and other cellular structures [64]. Currently, there is extensive research on the mechanism by which autophagy regulates Treg differentiation [65–68]. Autophagy contributes to Treg differentiation and the preservation of Treg function by regulating cellular metabolism [68]. Deletion of autophagy-regulated (Atg)7 and Atg5 in Tregs has been shown to decrease Foxp3 expression, leading to apoptosis. Additionally, inhibiting autophagy in Tregs can elevate cellular glycolysis level, which is the primary metabolic pathway in Th17. This metabolic shift ultimately results in impaired Treg differentiation and function [68, 69].

Similarly, Atg5 has been identified as a necessity for IL-17-producing Treg differentiation. In Treg specific Atg5-deficient mice (*Foxp3*^cre^*Atg5*^fl/fl^), the expression of IL-17-producing Tregs in the gut is nearly undetectable. However, further investigations are required to elucidate how Atg5 regulates IL-17-producing Treg differentiation and the role of glycolysis in this process [13]. Techniques such as metabolomics may provide valuable insights into the metabolic regulatory mechanisms governing IL-17-producing Treg differentiation.

### Promotive role of Wnt/β-catenin signaling pathway in IL-17-producing Treg differentiation

Previous studies have affirmed the regulatory role of the Wnt/β-catenin signaling pathway in Treg and Th17 differentiation [70–74]. When Wnt is inactive, a cytoplasmic scaffold protein (Axin), casein kinase 1 alpha (CK1α), glycogen synthase kinase-3 beta (GSK-3β), and the colorectal adenomatous polyposis gene (APC) form a degradation complex that targets β-catenin for degradation in T cells, maintaining it at a low level [75]. This, in turn, enables T-cell factor 1 (TCF-1, a transcription factor that binds to activated β-catenin in the nucleus) and Foxp3 to bind jointly to the enhancer region of Th17-related genes [76]. This reduces the chromatin accessibility of Th17-related genes, thereby restraining the expression of genes associated with Th17 differentiation and promoting Treg differentiation [77–79]. Conversely, in the presence of Wnt protein ligands, Wnt becomes activated and forms a co-receptor complex with the Frizzled protein (Fzd) and low-density lipoprotein receptor 5/6 (LRP5/6). This complex blocks the formation of the Axin/CK1α/GSK-3β/APC complex, thus inhibiting β-catenin degradation and leading to its accumulation in the cytoplasm. GSK-3β and CK1α also induce β-catenin dephosphorylation by phosphorylating LRP5/6 [80, 81]. This results in the translocation of β-catenin from the cytoplasm into the nucleus, where it binds to the transcription factor TCF-1/lymphoid enhancer factor 1 (LEF1), ultimately inducing the transcription of RORγT and other genes and promoting Th17 differentiation [82–85].

In a mouse model, stable expression of β-catenin in Tregs (*Foxp3*^YFP−Cre^*Ctnnb1*^fl(ex3)^ mice) significantly increased the expression of RORγT in Tregs, leading to a decrease in Treg immunosuppressive activity. This suggests that activation of the Wnt/β-catenin signaling pathway may serve as a crucial mechanism promoting IL-17-producing Treg differentiation [86–88]. Subsequent investigations employed Foxp3-Chip-Seq and ATAC sequencing technology to shed light on the mechanism by which β-catenin promotes IL-17-producing Treg differentiation through epigenetic pathways. Compared to their wild-type counterparts, *Foxp3*^YFP−Cre^*Ctnnb1*^fl(ex3)^ mice exhibited significantly enhanced chromatin accessibility in genes related to Th17 differentiation (genes inhibited by TCF-1-Foxp3 co-binding) in Tregs. These findings imply that Wnt/β-catenin signaling pathway activation in Tregs disrupts the inhibitory TCF-1-Foxp3 co-binding, enhancing chromatin accessibility of Th17-related genes and promoting the expression of genes associated with Th17 differentiation (Fig. 1) [87].

### Maintenance of IL-10 expression in IL-17-producing Treg by HIF-1α

The effects of HIF-1α on IL-17 and IL-10 expression in T cells have been well-documented [89–95]. Increased glycolysis is observed during CD4^+^ T cell differentiation into Th17 [96–98]. HIF-1α, activated under hypoxia, enhances the expression of glycolytic-related genes in cells, thereby promoting Th17 differentiation [99, 100]. However, HIF-1α in Th1 cells can induce IL-10 production under hypoxic conditions, conferring a certain degree of immunosuppressive function to Th1 [101].

Given the dual regulatory potential of HIF-1α on T cells, its impact on IL-17-producing Treg differentiation and function has been studied. It has been confirmed that HIF-1α is one of the factors promoting IL-10 production in IL-17-producing Tregs [102]. High levels of HIF-1α are expressed in IL-17-producing Tregs within the lamina propria of the intestinal mucosa. Deletion of *Hif1a* in IL-17-producing Tregs leads to decreased IL-10 production, although it does not affect RORγT expression. Through Chip-Seq analysis, it was discovered that HIF-1α can bind to the *Il10* gene promoter region, as well as 26 kb upstream in the IL-17-producing Tregs distal enhancer region. Additionally, the RNA-binding protein DEAD box helicase 5 (DDX5) serves as a transcriptional corepressor by binding to the intronic region of *Hif1a* exon 1. This induces the disassembly of the R-loop of *Hif1a*, impedes the accumulation of RNA Pol2, and hinders histone H3K4 trimethylation on *Hif1a*. Consequently, *Hif1a* transcription is repressed, inhibiting HIF-1α-induced IL-10 production in IL-17-producing Tregs (Fig. 1) [102].

### Multiple roles of gut microbiota in regulating IL-17-producing Treg differentiation

Numerous studies have underscored the pivotal influence of gut microbiota and its metabolites on the differentiation of Treg and Th17 [103–105]. Certain bacteria such as *Lactobacillus reuteri* [106, 107], *Bacteroides fragilis* [108–110], *Bacteroides thetaiotaomicron* [111], *Clostridium* [112–115], and *Faecalibacterium prausnitzii* [116–119] have been shown to promote Treg differentiation within the intestine. They play a crucial role in maintaining intestinal immune homeostasis and inhibiting the occurrence of inflammatory responses. Conversely, segmented filamentous bacteria [120–122] and *Bifidobacterium adolescentis* [123] have the unique ability to induce intestinal Th17 differentiation. Metabolites generated by the intestinal flora constitute one of the mechanisms by which they regulate Treg and Th17 differentiation [124, 125]. Of particular note are short-chain fatty acids (SCFAs), significant metabolites produced by the microbial breakdown of cellulose (e.g., acetate, propionate, and butyrate) [126, 127]. SCFAs have been extensively studied for their role in modulating Treg and Th17 differentiation [128–132]. Through various pathways including G-protein-coupled receptor signaling and histone deacetylase (HDAC) modulation, SCFAs can induce Treg differentiation while inhibiting Th17 differentiation [133–135]. Furthermore, gut microbiota impacts Treg and Th17 differentiation by modulating bile acid metabolism, a subject extensively reviewed by Su et al. in the latest publication in *Frontiers in Immunology* 2023 [125].

Notably, gut microbiota also plays a crucial role in IL-17-producing Treg differentiation. Comparative analyses of mice raised in a specific-pathogen-free (SPF) environment and germ-free (GF) environment have revealed a significant reduction in IL-17-producing Treg differentiation in GF mice compared to SPF mice. Additionally, IL-17-producing Treg levels in the intestines of GF mice increased following fecal transplantation from healthy humans. Subsequent investigations identified *Clostridium ramosum* as an inducer of IL-17-producing Treg differentiation in the gut of GF mice, whereas *Peptostreptococcus magnus* failed to elicit such a response [15, 136]. Due to its vital role in maintaining immune tolerance towards gut microbiota, some studies have referred to IL-17-producing Treg as “microbiota-dependent RORγT^+^Foxp3^+^ Treg” [15, 137].

*Akkermansia muciniphila*, through Toll like receptor 4 (TLR4) signaling, induces intestinal IL-17-producing Treg differentiation to preserve intestinal homeostasis [137]. TLR4, as an immune receptor, stands as a pivotal molecule essential for shaping colonic ecology and maintaining intestinal homeostasis [138]. Notably, TLR4-knockout mice (*Tlr4*^−/−^) exhibited disrupted intestinal homeostasis, a marked reduction in *Akkermansia muciniphila* abundance, and decreased IL-17-producing Treg ratios within the gut when compared to wild-type mice. In this context, wild-type mice could induce IL-17-producing Treg differentiation through *Akkermansia muciniphila* transplantation, whereas *Tlr4*^−/−^ mice displayed no significant alterations in intestinal IL-17-producing Treg levels before and after *Akkermansia muciniphila* transplantation [137]. Moreover, beyond inducing IL-17-producing Treg differentiation, gut microbiota can also curtail excessive IL-17-producing Treg differentiation, thereby maintaining intestinal immune homeostasis. Tryptophan metabolites produced by the intestinal flora can inhibit the excessive differentiation of IL-17-producing Treg in the intestine through activation of the AhR [139]. Tryptophan can be metabolized by the intestinal flora into compounds such as indole and its derivatives, including indole lactic acid, indole acrylic acid, and Indole-3-carbinol (I3C) [140]. These indole derivatives, by activating AhR in various immune cells, regulate immune responses effectively [141, 142]. Notably, the ratio of IL-17-producing Tregs in the intestinal lamina propria (si-LP) and mesenteric lymph nodes (mLN) significantly increased in mice subjected to a tryptophan-deficient diet (TDD). Moreover, the TDD diet failed to alter the intestinal IL-17-producing Treg levels after the removal of intestinal flora with antibiotics. However, the administration of the AhR ligand I3C in TDD diet mice effectively inhibited the excessive differentiation of IL-17-producing Tregs and preserved intestinal homeostasis (Fig. 1) [139].

### Induction of IL-17-producing Treg differentiation by different types of antigen-presenting cells (APC)s

APCs encompass various cell types, including dendritic cells (DCs), mononuclear phagocytes, and B cells, among others. These cells play a pivotal role in capturing and processing antigens, subsequently presenting them to T cells to initiate specific immune responses [143, 144]. Notably, among APCs, DCs exhibit the most robust antigen-presenting capabilities [145]. Extensive research has elucidated the regulatory influence of DCs on the differentiation of Treg and Th17 [146–150]. Activation of interferon regulatory factor 4 (IRF4) in human intestinal DCs has been found to induce the production of IL-6 and IL-23, thereby promoting T cell secretion of IL-17 [151, 152]. Furthermore, DCs can induce Th17 differentiation through the production of Integrin αVβ8, which activates TGF-β [153]. Langerhans cells (LCs) residing in the skin have demonstrated the ability to induce IL-6 production by activating Dectin-1, particularly in response to stimuli from microorganisms like *Candida albicans* or *Staphylococcus aureus*. This IL-6 production subsequently promotes Th17 differentiation [154–157]. Additionally, specific DC subsets can drive Treg differentiation by producing retinoic acid (RA) [158], indoleamine 2, 3-dioxygenase (IDO) [159, 160], IL-10 [161], and TGF-β [162]. This is facilitated through the presence of immunomodulatory molecules such as programmed death-ligand (PD-L)1, PD-L2 [163, 164], B and T lymphocyte attenuator (BTLA) [165], and ICOS ligand (ICOSL) [166] on the DC cell membrane. A comprehensive review of the regulatory mechanisms employed by DCs in Treg and Th17 differentiation has been conducted by Yin et al. [150].

On September 7, 2022, *Nature* published three articles detailing the mechanisms behind APC-induced IL-17-producing Treg differentiation, a process crucial for establishing tolerance to gut microbiota in the body [21, 167, 168]. These studies collectively revealed the existence of distinct APC-induced IL-17-producing Treg differentiation pathways in neonatal and adult mice. At birth, a novel APC termed “Thetis cells” was identified as responsible for inducing IL-17-producing Treg differentiation, thus facilitating neonatal mouse tolerance to gut microbiota. Single-cell sequencing analyses indicated a significant surge in IL-17-producing Treg numbers within the long intestinal lymph nodes of mice ten days post-birth. This IL-17-producing Treg differentiation was found to be regulated by APCs expressing RORγT. Subsequent isolation and analysis of RORγT-expressing APCs (RORγT^+^MHC^+^) using single-cell sequencing and single-cell ATAC sequencing (scATAC-Seq) led to the identification of Thetis cells as cells as a novel APC subset. Thetis cells were further categorized into four subtypes based on gene expression patterns. Specifically, Type I and III Thetis cells expressed the thymus epithelial cell marker autoimmune regulator (AIRE), Type II Thetis cells expressed CD11c and CCR6, and Type IV Thetis cells expressed CD11c and CD11b. Comparative gene expression analysis of these four Thetis cell subtypes with DCs and group 3 innate lymphoid cell (ILC3)s, as well as an examination of Thetis cell ontogeny, confirmed their unique identity. Since the TGF-β pathway plays a pivotal role in inducing IL-17-producing Treg differentiation, it was noteworthy that Type IV Thetis cells expressed multiple genes associated with the TGF-β pathway, including *integrin alpha V* (*Itgav*) and *integrin beta 8* (*Itgb8*). Notably, *Itgb8* was uniquely expressed by Type IV Thetis cells among RORγT^+^ cells. Conditional removal of DC and ILC3 in vivo did not affect IL-17-producing Treg differentiation in neonatal mice, indicating that Type IV Thetis cells are essential for inducing IL-17-producing Treg at birth. This assertion was substantiated by creating *Itgb8*-specific gene knockout (*Itgb8*^*ΔRORγT*^) mice, which exhibited significantly lower post-birth IL-17-producing Treg levels compared to wild-type controls. Furthermore, Thetis cells were found to be abundant within three weeks post-birth but gradually disappeared by the sixth week, affirming the role of Type IV Thetis cells as the APCs responsible for IL-17-producing Treg induction during neonatal birth [21]. Another study reported that in the adult gut, ILC3s play a crucial role in presenting antigens from intestinal microbiota to Tregs via MHC-II molecules on their surface. Simultaneously, they secrete alpha V integrin to induce IL-17-producing Treg differentiation, thus enabling tolerance to *Helicobacter hepaticus*. Single-cell sequencing revealed that RORγT^−^ expressing cells in the follicular region of adult mouse lymph nodes primarily consisted of ILC3s and IL-17-producing Tregs. Specific deletion of MHC-II in mouse ILC3s (*MHCII*^*ΔILC3*^ mice) resulted in a substantial reduction in gut IL-17-producing Tregs. Moreover, when *Helicobacter hepaticus* was transplanted into *MHCII*^*ΔILC3*^ mice, IL-17-producing Treg differentiation was not induced, leading to the loss of intestinal immune tolerance and the emergence of enteritis. Inhibition of ILC3 secretion of αV integrin effectively nullified the induction of IL-17-producing Treg differentiation by ILC3s [168]. It’s noteworthy that while *Helicobacter hepaticus* can induce IL-17-producing Treg differentiation and immune tolerance, excessive proliferation of *Helicobacter hepaticus* can also trigger Th17 responses, leading to intestinal inflammation [169]. Another study elucidated the mechanism by which *Helicobacter hepaticus* induces differentiation of IL-17-producing Treg or Th17. It was confirmed that *Helicobacter hepaticus* can trigger the differentiation of IL-17-producing Tregs in APCs, including ILC3s and Janus cells, which express RORγT. This process is facilitated by the chemokine CCR7 and the TGF-β agonist αV integrin. Colonization of *Helicobacter hepaticus* in wild-type mice not only induces IL-17-producing Treg differentiation but also establishes immune tolerance to *Helicobacter hepaticus*. However, when *Helicobacter hepaticus* lacks antigen-presenting ability (*MHCII*^*ΔCD11c*^ mice), it fails to induce IL-17-producing Treg differentiation upon colonization, leading to Th17-mediated intestinal inflammation. This observation underscores the crucial role of APCs in facilitating *Helicobacter hepaticus*-induced IL-17-producing Treg differentiation. Further investigations utilizing Cellular Indexing of Transcriptomes and Epitopes by Sequencing (CITE-seq) identified specialized APC cells, namely ILC3s expressing RORγT and MHC-II, as well as Janus cells (RORγT^+^ APCs), which actively promote IL-17-producing Treg differentiation. In mice lacking antigen-presenting capabilities in ILC3s and Janus cells (*MHCII*^*ΔRORγT*^), colonization with *Helicobacter hepaticus* fails to induce IL-17-producing Treg differentiation. This model revealed that CCR7 and αV integrin are essential for initiating IL-17-producing Treg differentiation through RORγT^+^ APCs. Strikingly, both *CCR7*^*ΔRORγT*^ and *Itgav*^*ΔCD11c*^ mice, which lack these necessary components, are unable to induce IL-17-producing Treg differentiation in response to *Helicobacter hepaticus*. Consequently, Th17-mediated intestinal inflammation ensues [167]. These two studies collectively shed light on the intricate mechanism by which *Helicobacter hepaticus* elicits resistance through the induction of IL-17-producing Treg differentiation mediated by antigen-presenting ILC3s (Fig. 1).

### Other factors (costimulatory molecules, zinc finger protein and microRNAs) involved in IL-17-producing Treg differentiation

T cell activation necessitates the engagement of dual signals, comprising the interaction between MHC molecules on the APC surface and TCR (the first signal), as well as the activation of co-stimulatory molecules (the second signal). IL-17-producing Treg expresses a range of co-stimulatory/co-inhibitory molecules that exert both positive and negative regulatory effects on IL-17-producing Treg differentiation and proliferation. In the presence of MHC-II, the co-stimulatory molecules CD28 and ICOS promote IL-17-producing Treg differentiation, whereas the co-inhibitory molecule CTLA-4 inhibits IL-17-producing Treg proliferation. The application of CD28 and ICOS neutralizing antibodies can decrease the IL-17-producing Treg ratio in the lamina propria of the mouse intestine, whereas CTLA-4 neutralizing antibody can enhance IL-17-producing Treg proliferation [170].

Zinc finger protein 362 (ZFP362), a member of the Cys2/His2 (C2H2) zinc finger protein family, possesses transcriptional factor and transcriptional regulatory factor activities [171]. While ZFP362 expression is increased in Th17 and can interact with RORγT to promote Th17 differentiation [172], recent investigations have unveiled its regulatory role in IL-17-producing Treg differentiation. Notably, the level of IL-17-producing Tregs in the mesenteric lymph nodes of *Zfp362*^−/−^ mice surpasses that in wild-type mice [173]. Nevertheless, further exploration is warranted to fully elucidate the mechanisms by which ZFP362 regulates IL-17-producing Treg differentiation.

MicroRNAs (miRNAs), a class of non-coding RNAs approximately 20–25 bp in length, have the capability to bind to the 3’ UTR of specific mRNA molecules, subsequently leading to mRNA degradation or the inhibition of translation. Extensive research has elucidated the role of miRNAs in the differentiation of Treg and Th17, as comprehensively reviewed by Liu et al. [174]. It has also come to light that certain miRNAs possess the potential to regulate IL-17-producing Treg differentiation. For instance, the transfection of miR-182 has been shown to promote the differentiation of Jurkat cells into IL-17-producing Treg [54].

### IL-10-producting TH17

The instability and plasticity of Th17 cells enable their transformation from a pro-inflammatory phenotype, characterized by the secretion of pro-inflammatory cytokines like IL-17, into an anti-inflammatory phenotype that produces immunosuppressive factors, such as IL-10, in specific environmental conditions [175]. The discovery of IL-10-producing Th17 cells, referred to as IL-10-producing Th17, was initially reported in a study published in *Nature* in 2011 [26]. The anti-CD3-induced colitis model serves as a research tool to investigate intestinal immune tolerance. Typically, mice develop transient colitis after anti-CD3 stimulation, followed by gradual recovery [176]. When the mucosal immune system’s tolerance to gut microbiota diminishes, mice stimulated with anti-CD3 displayed prolonged inflammatory responses in the gut [11, 177, 178]. Interestingly, the authors observed that anti-CD3 stimulation induced a substantial activation of Th17 cells in the intestine. Surprisingly, these Th17s did not undergo apoptosis as expected after the resolution of intestinal inflammation but instead persisted. Upon isolation, it was evident that these Th17s not only expressed IL-17 but also exhibited high levels of IL-10 expression, demonstrating significant immunosuppressive activities in vitro [26]. A subsequent study published in *Nature* in 2012 revealed that repeated stimulation of T cells by *Staphylococcus aureus* resulted in their differentiation into Th17 with IL-17 production. However, upon continued stimulation with *Staphylococcus aureus*, these cells shifted their cytokine profile towards IL-10 secretion, indicating that *Staphylococcus aureus* stimulation can induce Th17 to acquire anti-inflammatory properties. Notably, the IL-10-producing capability of these Th17s diminished, and they reverted to secreting only IL-17 after the removal of *Staphylococcus aureus* stimulation [26]. Moreover, Th17 has the potential to differentiate into Treg and Treg type 1 (Tr1) [179, 180]. In a study published in *Nature* in 2015, it was further confirmed that IL-10-producing Th17 represents an intermediate state of Th17s transitioning into Tr1s. This study showed that Th17 could be stimulated to become IL-10-producing Th17, co-expressing both IL-10 and IL-17 A. Subsequently, IL-10-producing Th17 lose their IL-17 A expression and exclusively produce IL-10, referred to as Tr1^exTh17^ cells. Tr1^exTh17^ cells exhibit gene expression patterns and immunological characteristics highly similar to those of Tr1s [25].

### The characteristics and physiological function of IL-10-producing Th17

**Fig. 2:**
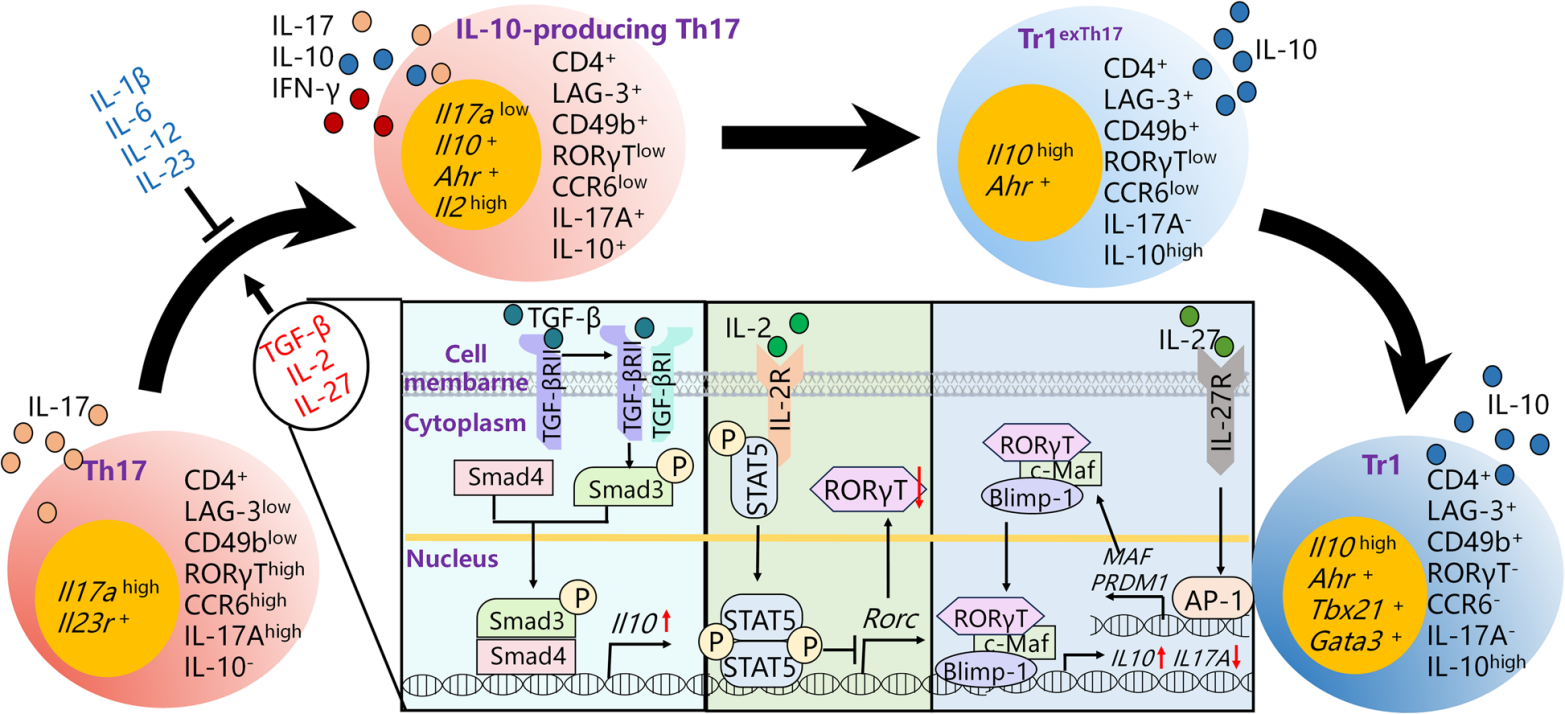
Regulation of IL-10-producing Th17 differentiation. Th17 cells could convert into IL-10-producing Th17, co-expressing both IL-10 and IL-17 A. Subsequently, IL-10-producing Th17 lose their IL-17 A expression and exclusively produce IL-10, referred to as Tr1^exTh17^ cells. Tr1^exTh17^ cells could further differentiate into Tr1s. TGF-β, IL-2 and IL-27 have been shown to induce IL-10-producing Th17 differentiation, whereas IL-1β, IL-6, IL-12 and IL-23 could inhibit IL-10-producing Th17 differentiation

It has been established that IL-10-producing Th17s are capable of simultaneously secreting IL-10, IL-17, and IFN-γ [26]. When comparing the gene expression and cell surface markers of IL-10-producing Th17s with that of Th17, Tr1^exTh17^, Treg, and Tr1, it becomes evident that IL-10-producing Th17s exhibit low expression of *Rorc* but express *Il17a*. In contrast to Tr1^exTh17^ cells, IL-10-producing Th17s express *Il17a*, and unlike Treg cells, IL-10-producing Th17s do not express *Foxp3*. Furthermore, IL-10-producing Th17s express certain levels of Tr1 markers, such as lymphocyte activating 3 (LAG-3), as well as integrin alpha 2 (CD49B) and aryl hydrocarbon receptor (AhR). Notably, the gene expression pattern of Tr1^exTh17^ cells is more similar to that of IL-10-producing Th17 and Tr1 than to Th17s, further substantiating the notion that Tr1^exTh17^ cells represent an advanced form of IL-10-producing Th17s transitioning into Tr1s (Fig. 2) [25]. Given that IL-10-producing Th17s predominantly reside in the gut, one study investigated their distribution compared to Tr1^exTh17^ and Th17 in various parts of the gastrointestinal tract. The findings revealed that over 60% of IL-10-producing Th17s are distributed in the ileum, with around 30% residing in Peyer’s patches, and the remaining 10% distributed in the jejunum, duodenum, and colon [27]. Flow cytometry is widely used to investigate IL-10-producing Th17 in multiple tissues. The detailed information for investigating IL-10-producing Th17 is shown in Table 1.

In physiological conditions, IL-10-producing Th17s play a crucial role in maintaining immune tolerance to the gut microbiota. In comparison to wild-type mice, mice with specific *Il10* gene knockout in Th17 (*Il17a*^cre^*Il10*^fl/fl^ mice) exhibited significantly elevated expression of pro-inflammatory cytokines such as *Il17f*, *Il1b*, *interferon gamma* (*Ifng)*, *chemokine (C-X-C motif) ligand 11* (*Cxcl1*), and *tumor necrosis factor a* (*Tnfa*) in the gut, along with an increase in Th1 cell levels. Additionally, IL-10-producing Th17s have been identified as key players in maintaining immune homeostasis in the intestinal mucosa. Moreover, *Il17a*^cre^*Il10*^fl/fl^ mice subjected to anti-CD3 stimulation developed severe intestinal inflammation in comparison to their wild-type counterparts [27].

### Regulatory factors involved in IL-10-producing Th17 differentiation

There is currently a limited body of literature exploring the regulatory mechanisms governing IL-10-producing Th17 differentiation. Given that IL-10-producing Th17 represents an intermediate state between Th17 and Tr1, it is pertinent to consider pathways known to regulate the transformation from Th17 to Tr1, including the TGF-β signaling pathway and certain cytokines, which have been identified as inducers of IL-10-producing Th17 differentiation.

### Induction of IL-10-producing Th17 cell differentiation by TGF-β signaling pathway

TGF-β plays a pivotal role as a regulatory factor in guiding the differentiation of CD4^+^ T cell into both Th17 and Treg [59]. In the context of Th17, TGF-β interacts with TGF-β receptor (TGFβR) II on the cell surface, triggering the secretion of IL-10 by Th17. Notably, Th17 with specific *Tgfbr2* knockout exhibited a significant reduction in IL-10 production compared to their wild-type counterparts [27]. TGFβRII is a transmembrane protein comprised of 567 amino acids, featuring an extracellular domain, a transmembrane domain, and an intracellular domain. TGF-β binding to the extracellular domain of TGFβRII induces phosphorylation of the extracellular domain. Subsequently, the phosphorylated TGFβRII-TGF-β complex activates TGFβRI on the cell membrane, thereby promoting Smad3 phosphorylation. Phosphorylated Smad3 forms a transcription complex with Smad4, facilitating the transcription of the *Il10* gene [59] (Fig. 2). Mice with *Smad4*-specific knockout in Th17 cells exhibited significantly reduced IL-10 production in Th17. Moreover, using a dual-luciferase reporter gene technique, it was confirmed that the activation of Smad3/4 in Th17 binds to the promoter region of the *Il10* gene, thereby inducing IL-10 secretion by Th17 [27]. Additionally, TGF-β can induce IL-10 secretion by activating AhR in Th17 [25].

### Promotive role of IL-27 and suppressive roles of IL-1β, IL-6, IL-12, IL-23, IL-2 in IL-10-producing Th17 cell differentiation

The differentiation of IL-10-producing Th17 is subject to regulation by a range of cytokines. Notably, IL-1β, IL-6, IL-12, and IL-23 have been identified as inhibitors of IL-10 production by IL-10-producing Th17. Among these, IL-1β exerts the most pronounced inhibitory effect on IL-10 production by IL-10-producing Th17, potentially linked to its activation of the nuclear factor-kappa B (NF-κB) signaling pathway [26, 181]. IL-2 inhibits RORγT expression in Th17 through activating signal transducer and activator of transcription 5 (STAT5) [26, 182]. Conversely, IL-27 has been shown to promote IL-10 production by IL-10-producing Th17 [29]. IL-27 binds to IL-27 receptor subunit alpha (IL-27Ra) on the surface of Th17, activating activator protein-1 (AP-1) family transcription factors, which, in turn, promote the expression of *musculoaponeurotic fibrosarcoma* (*Maf*) [183]. Additionally, IL-27 induces *B-lymphocyte-inducing maturation protein 1* (*Blimp1*) expression through early growth response gene 2 (Egr-2) Subsequently, the Maf/RORγT/Blimp-1 complex forms, inhibiting *Il17* expression driven by RORγT alone. Moreover, the Maf/RORγT/Blimp-1 complex translocates into the nucleus, promoting IL-10 production by Th17. Furthermore, studies have corroborated that IL-27 counteracts the inhibitory effects of IL-12 on IL-10-producing Th17 differentiation through Blimp-1 (Fig. 2) [29].

### The roles of IL-17-producing Treg and IL-10-producing TH17 in disease

The imbalance between the Treg and Th17 ratio plays a pivotal role in the pathogenesis of a wide a wide array of inflammatory diseases [184], tumors [185], autoimmune disorders [186, 187], and metabolic conditions [188–190]. A sour understanding of the contributions of IL-17-producing Treg and IL-10-producing Th17 in these diseases continues to expand, their associations with conditions such as inflammatory bowel disease (IBD), cancer, nephritis, autoimmune disorders, and others are gradually being unveiled.

### IBD

As a vital component of the intestinal mucosal immune system, the equilibrium between Treg and Th17 stands as a pivotal immunological mechanism in the onset of IBD [191, 192]. Both clinical observations and animal experiments have consistently shown that, in comparison to healthy controls, IBD patients exhibit an elevated proportion of Th17 in the peripheral blood and gut, which secrete substantial quantities of pro-inflammatory cytokines. Concurrently, there is a decrease in Treg proportion or a relative deficiency in Treg function, resulting in a reduction or relative deficiency of immunosuppressive factors like IL-10. This imbalance, characterized by a higher Treg/Th17 ratio, fosters the development of intestinal inflammatory reactions [193, 194]. The mechanisms underlying the Treg/Th17 imbalance in IBD have been thoroughly reviewed by Iacomino et al. [195]. Given their significant presence in the gut, the relationship between IL-17-producing Treg and IL-10-producing Th17 and IBD has attracted extensive discussion.

### Promotive role of IL-17-producing Treg in the development of IBD

In peripheral blood and intestinal inflammatory areas of IBD patients, the IL-17-producing Treg ratio was found to be increased in comparison to healthy controls. In particular, IL-17-producing Treg in the peripheral blood of IBD patients was found to possess pro-inflammatory properties, associated with sustained activation of β-catenin cells [87]. Furthermore, recent single-cell sequencing revealed a substantial infiltration of IL-17-producing Treg in the colonic mucosa of patients with ulcerative colitis (UC) and Crohn’s disease (CD) [24]. Compared to the remission period of UC patients, the peripheral blood IL-17-producing Treg levels in patients in the active phase were significantly elevated and positively correlated with the levels of pro-inflammatory factors such as IL-21 in the serum. Drug therapy-induced remission resulted in a significant decrease in peripheral blood IL-17-producing Treg levels in patients with active-phase UC [23]. In a mouse model of Giardia-induced diarrhea, the proportion of intestinal IL-17-producing Tregs increased, thereby enhancing mouse susceptibility to Giardia [196]. Tryptophan deficiency in the diet may lead to excessive proliferation of intestinal IL-17-producing Tregs and promote the progression of intestinal inflammation [139].

### The amelioration of IBD by promoting IL-17-producing Treg differentiation

One study found that despite the increased proportion of IL-17-producing Tregs in the ileum, colon, and rectum of patients with Crohn’s disease compared to healthy individuals, these cells secrete IL-17. However, in vitro experiments have demonstrated their significant immunosuppressive activity [197]. Transplantation of *Rag*^−/−^ mice with IL-17-producing Tregs can mitigate dextran sodium sulfate (DSS)-induced colitis [17]. The transplantation of healthy human feces can ameliorate DSS-induced UC in GF mice and increase the number of IL-17-producing Tregs, illustrating that gut probiotics can enhance UC by promoting IL-17-producing Treg differentiation [136]. Moreover, mice lacking Arkadia or Atg5-mediated differentiation of IL-17-producing Tregs display intestinal inflammation [13, 61]. It has also been reported that in the context of inflammatory bowel disease, ILC3s can alleviate intestinal inflammation by promoting IL-17-producing Treg differentiation [168]. Environmental enteric dysfunction (EED) is an intestinal inflammatory disease that can lead to stunted growth and a weakened response to oral vaccines in children [198, 199]. A study found that in an EED model, the level of IL-17-producing Tregs in the gut significantly increased, which suppressed intestinal inflammation and improved stunted growth caused by EED. However, IL-17-producing Tregs also suppressed CD4^+^ T cells in the gut that respond to oral vaccines, resulting in reduced vaccine effectiveness. Mice lacking IL-17-producing Tregs (*Foxp3*^cre−ETR2^*Rorc*^fl/fl^) displayed more severe stunting after the induction of EED compared to wild-type controls, but they exhibited a stronger response to oral vaccines [200].

### Anti-inflammatory role of IL-10-producing Th17 in IBD

Notably, IL-10-producing Th17s were initially found to possess anti-inflammatory properties and induce tolerance to secondary gut bacterial infections, such as *Staphylococcus aureus*, thereby ameliorating intestinal inflammation, as reported in *Nature* [25]. Similarly, in a mouse model of colitis induced by CD3 monoclonal antibody + tamoxifen, the induction of IL-10-producing Th17 was found to inhibit inflammatory reactions [25]. However, there are currently conflicting conclusions regarding the relationship between IL-17-producing Tregs and IBD.

These findings suggest that IL-17-producing Tregs may have multifaceted roles in both promoting and suppressing inflammation in IBD. However, it is evident from these contrasting conclusions that different immune properties may be mediated by different pathways. For instance, β-catenin [87] and tryptophan deficiency [139] can augment the pro-inflammatory activity of IL-17-producing Tregs, while Arkadia [61], Atg5 [13], ILC3s [168], and certain probiotics can enhance the anti-inflammatory activity of IL-17-producing Tregs. Understanding the mechanisms governing the pro-inflammatory/anti-inflammatory immune activities of IL-17-producing Tregs represents a future research direction aimed at elucidating their dual role in IBD. Although there is evidence supporting the anti-inflammatory properties of IL-10-producing Th17 in IBD, it is also imperative to investigate whether IL-10-producing Th17 may exhibit pro-inflammatory activity in IBD and under what circumstances such activity may occur.

### Tumor

The infiltration of Treg and Th17 into the tumor microenvironment can exert influence on tumor progression through various mechanisms [201]. The impact of Th17 on tumors is bidirectional. IL-17, secreted by Th17, contributes to the immunosuppressive environment within tumors by inducing vascular endothelial growth factor (VEGF) expression and promoting the differentiation of myeloid-derived suppressor cells (MDSCs) in the tumor microenvironment. This inflammatory response is often referred to as promoting a pro-tumor inflammatory response [202–205]. Conversely, IL-17 has also been reported to enhance the recruitment of anti-tumor immune cells within tumor tissues, consequently elevating IFN-γ levels within the tumor microenvironment, which plays a role in inhibiting tumor progression [206]. This contrasting effect may be influenced by the type and stage of the tumor, as reviewed by Gorczynski [207]. Tregs are generally believed to drive tumor development by suppressing immune responses, making targeted inhibition of Treg differentiation an important approach in tumor treatment [208–211]. Currently, there is evidence suggesting that IL-17-producing Treg and IL-10-producing Th17 are associated with tumor development.

### Induction of cancer-initiating cell differentiation and immunosuppression by IL-17-producing Treg

In comparison to healthy controls, the quantity of IL-17-producing Tregs in tumor tissues of colon cancer and breast cancer patients has been found to increase [54, 212, 213]. Studies have verified that IL-17-producing Tregs can induce the differentiation of colon cancer-initiating cells by secreting IL-17. After co-culturing IL-17-producing Tregs isolated from colon cancer patients’ tumors with Sphere cells derived from normal human bone marrow, Sphere cells exhibited increased expression of multiple colon cancer-initiating cell-related phenotypes, such as CD133, CD44s, CD166, epithelial cell adhesion molecule (EpCAM), and aldehyde dehydrogenase 1 (ALDH1). Notably, the addition of recombinant IL-17 A neutralizing antibody to the co-culture system impeded the inductive effect of IL-17-producing Tregs on colon cancer-initiating cell differentiation [22]. Furthermore, IL-17-producing Treg has also been shown to involve in the immunosuppression within the tumor. Inhibition of IL-17-producing Treg differentiation can enhance anti-tumor immune responses [212].

### Promotive role of IL-17-producing Treg in the development of colitis-associated colorectal cancer (CAC)

Given the diverse immune activities of IL-17-producing Treg and IL-10-producing Th17, coupled with their role as intermediaries in the conversion between Treg and Th17, the involvement of IL-17-producing Treg and IL-10-producing Th17 in the carcinogenesis of colon tumors has garnered considerable attention. Colon polyposis serves as the primary precancerous lesion leading to colon cancer, and IL-17-producing Tregs have been observed to be extensively infiltrated in colon polyposis in the mouse model of *Adenomatous polyposis coli* (*Apc*^Δ^). Inhibition of IL-17-producing Treg differentiation in these mice can reduce the incidence of colon polyposis [212]. Similarly, research has shown that IL-17-producing Tregs are highly expressed in the spleen and tumor tissues of *Apc*^Δ^ mice treated with DSS to induce IBD-associated CAC. These IL-17-producing Tregs express elevated levels of cell proliferation-related phenotypes, such as CD44 and Ki67 [87]. Subsequent studies have demonstrated that IL-17-producing Tregs promote the development of colorectal cancer by influencing DCs within the tumor microenvironment, resulting in heightened inflammatory responses and CAC. Moreover, mice with a conditional knockout of RORγT in Tregs (*Foxp3*^GFP−cre^*Rorc*^fl/fl^ mice) exhibit lower susceptibility to colorectal cancer induction by azoxythane (AOM)/DSS treatment compared to wild-type mice. This can be attributed to the low expression of CTLA-4 by IL-17-producing Tregs, which leads to Forkhead box O 3 (FoxO3) inactivation in DCs and the activation of the IL-6 production-STAT3 signaling pathway in tumor cells, ultimately promoting their proliferation. The activation of STAT3 subsequently induces tumor cell proliferation. Expression of STAT3 and IL-6 by DCs is diminished in *Foxp3*^GFP−cre^*Rorc*^fl/fl^ mice, while CTLA-4 expression is elevated in Tregs compared to wild-type mice. Co-culturing DCs with Tregs from *Foxp3*^GFP−cre^*Rorc*^fl/fl^ mice results in reduced IL-6 expression in DCs, an effect that is abolished following FoxO3 knockdown or CTLA-4 neutralization antibody treatment. Silencing FoxO3 in *Foxp3*^GFP−cre^*Rorc*^fl/fl^ mice accelerates tumor development, accompanied by increased IL-6 and STAT3 expression within the tumor tissue [213].

### Increase of IL-10-producing Th17 differentiation in acute myeloid leukemia (AML) patients

A substantial presence of IL-10-producing Th17 has been observed in the peripheral blood of AML patients. In vitro experiments have revealed that the presence of IL-10-producing Th17 diminishes the immune response of T cells against bacterial infections. Induction of IL-10-producing Th17 differentiation can be accomplished by co-culturing peripheral blood CD33^+^ myeloid cells from AML patients with normal CD4^+^ T cells. However, this phenomenon is not observed with CD33^+^ myeloid cells from healthy individuals [28].

Although the aforementioned evidence collectively indicates the promotional effect of IL-17-producing Tregs on intestinal tumors, it is important to acknowledge that IL-17-producing Tregs can induce both pro-inflammatory reactions that promote tumorigenesis (pro-inflammatory properties) and immunosuppression that facilitates tumor evasion (immunosuppressive properties). These seemingly contradictory findings parallel the dual promoting and inhibitory effects of IL-17-producing Tregs observed in IBD. This underscores the importance of investigating the regulatory mechanisms governing IL-17-producing Tregs’ manifestation of both immune activation and inhibition, which will be a crucial avenue for researchers to better comprehend the functionality of IL-17-producing Tregs.

### Autoimmune diseases

The association between the disruption of the Treg and Th17 balance and autoimmune diseases has been the focus of extensive research. On one hand, the excessive differentiation of Th17 can trigger autoimmune reactions, while the relative scarcity of Treg results in the loss of immune tolerance within the body [214–217]. Multiple signaling pathways are implicated in the imbalance between Treg and Th17 in autoimmune diseases, as comprehensively reviewed by Lee [6]. IL-17-producing Tregs have also been closely linked to various autoimmune diseases, including rheumatoid arthritis (RA), autoimmune encephalomyelitis (EAE), systemic lupus erythematosus (SLE), and more. Available evidence suggests that suggests that IL-17-producing Tregs exhibit diverse roles in these diseases.

### Inhibition of RA inflammation by IL-17-producing Treg

The heightened infiltration of IL-17-producing Tregs in the joint cavity of collagen-induced arthritis (CIA) mice implies their involvement in RA-related inflammatory responses. Further investigations have revealed that IL-17-producing Tregs in the joint cavity of CIA mice express elevated levels of CD25, CTLA4, GITR, ICOS, and secrete substantial quantities of IL-10. Notably, these IL-17-producing Tregs also express CCR6, suggesting their potential migration to the joint cavity under the influence of CCR6. Mice from which IL-17-producing Tregs were removed (*Foxp3*^cre^*Rorc*^fl/fl^ mice) developed more severe RA compared to wild-type mice [18], and the injection of IL-17-producing Tregs resulted in increased IL-10 levels and diminished inflammatory responses in RA mice [218].

### Suppression of EAE autoimmune responses by IL-17-producing Treg

In the experimental autoimmune EAE model, induced by myelin oligodendrocyte glycoprotein (MOG) and complete Freund’s adjuvant (CFA), there is an increase in CCR6^+^ICOS^high^ IL-17-producing Tregs within lymph nodes. Isolation and subsequent culture of these CCR6^+^ICOS^high^ IL-17-producing Tregs with effector T cells revealed their capacity to inhibit effector T cell activation, suggesting their immunosuppressive activity. Additionally, in the Th17-mediated EAE model established in *Rag1*^−/−^ mice through the *Rag1*^−/−^ mice through the co-transfer of Th17 and CCR6^+^ICOS^high^ IL-17-producing Tregs, the latter effectively suppressed the autoimmune response induced by Th17 [20].

### Exacerbation of kidney damage in SLE by IL-17-producing Treg

In a Pristane-induced SLE mouse model, IL-17-producing Treg infiltration in kidney tissue gradually increased with disease progression. Additionally, after Pristane induction, *Foxp3*^cre^*Rorc*^fl/fl^ mice exhibited less kidney damage and decreased IL-17 levels compared to wild-type mice [16].

### Other diseases

IL-17-producing Treg has also been demonstrated to involve in the progression of nephrotoxic nephritis (NTN), type 2 diabetes mellitus (T2DM) and non-alcoholic steatohepatitis (NASH). Inducing IL-17-producing Treg differentiation has shown with a mitigating effect in crescentic glomerulonephritis. In a mouse model of NTN-induced crescentic glomerulonephritis, a significant increase in IL-17-producing Treg levels within renal tissue occurred four days prior to NTN induction, followed by a gradual decline. Further analysis revealed that IL-17-producing Tregs secrete both pro-inflammatory and anti-inflammatory factors, and transplantation of IL-17-producing Tregs into crescentic glomerulonephritis mice substantially improved kidney damage. Interestingly, the mechanism by which IL-17-producing Tregs alleviate kidney injury in crescentic glomerulonephritis mice differs from Treg cells [219]. However, a comprehensive understanding of how IL-17-producing Tregs ameliorate kidney injury in crescentic glomerulonephritis mice remains to be elucidated. Furthermore, there is an elevated IL-17-producing Treg ratio in the peripheral blood of patients with T2DM, and this ratio is positively correlated with body mass index (BMI) and glycosylated hemoglobin type A1c (HbA1c), implying the potential involvement of IL-17-producing Tregs in the progression of T2DM [220]. Elevated IL-17-producing Treg levels have also been detected in the liver tissues of NASH mice induced by a high-fat diet. Nevertheless, upon the removal of IL-17-producing Tregs, more severe liver fibrosis occurred in high-fat diet mice. Furthermore, the population of γδT cells expressing Th17 and IL-17 increased significantly within liver tissues. This suggests that IL-17-producing Tregs play a role in inhibiting liver inflammation and fibrosis. Interestingly, the enrichment of IL-17-producing Tregs in liver tissue appears to depend on the presence of intestinal flora, as their quantity decreased following antibiotic-induced removal of intestinal flora in NASH model mice [15].

IL-10-producing Th17 contributed to the progression of endometriosis (EMS). In patients with EMS, a notable increase in the number of IL-10-producing Th17 has been observed within the endometriotic milieu, and this elevation escalates as the disease progresses. Promotion of IL-10-producing Th17 differentiation exacerbates EMS. IL-27 has been identified as a key factor inducing IL-10-producing Th17 differentiation in EMS [29].

### The association between IL-17-producing Treg and IL-10-producing TH17 and Chinese traditional “Yin-Yang” theory

**Fig. 3:**
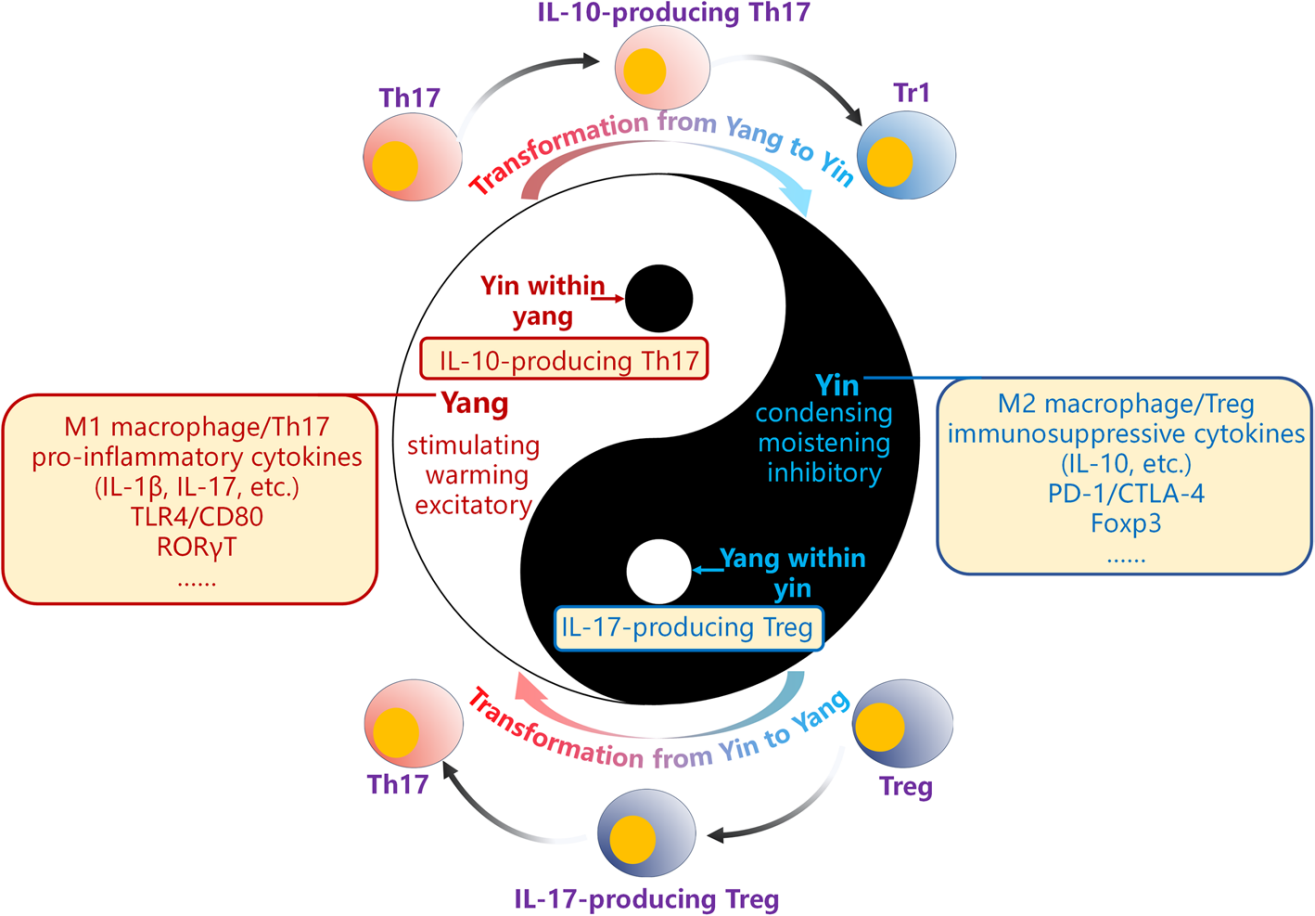
“Yin-Yang” theory and immune system. According to the opposing attributes of Yin and Yang, immune-activating cells and molecules, such as M1 macrophages, Th17s, and pro-inflammatory cytokines like IL-1β and IL-17, as well as immune activation-related molecules such as TLR4 and CD80, along with the transcription factor RORγT, are associated with Yang, represented by the white “fish”. Immunosuppressive cells and molecules such as M2 macrophages, Treg, immunosuppressive cytokines such as IL-10, inhibitory receptors PD-1 and CTLA-4, and the transcription factor Foxp3, are associated with Yin, represented by the black “fish”. Furthermore, Yin and Yang are mutually inclusive, represented by the black circle in Yang (Yin within Yang) and the white circle in Yin (Yang within Yin). Therefore, IL-10-producing Th17 can be viewed as a manifestation of Yang within Yin, whereas IL-17-producing Treg can be seen as Yin within Yang. Besides, the transformation of Yin and Yang are also reflected in the study of IL-17-producing Treg and IL-10-producing Th17 as IL-17-producing Treg and IL-10-producing Th17 represent intermediate forms in the transformation between Treg and Th17

Ancient Chinese philosophy includes the significant concept of “Yin-Yang” theory. In brief, Yin and Yang represent opposing aspects of phenomena. In medicine, Yin pertains to substances and functions with a condensing, moistening, and inhibitory effect on the body, often depicted as the black “fish.” Conversely, Yang encompasses substances and functions that exert a stimulating, warming, and excitatory influence, represented by the white “fish.” Yin and Yang interact in a mutually restraining and complementary manner, with transitions between them. Yin can transform into Yang, and vice versa, under specific conditions, such as when Yin/Yang substances increase to a certain point and overcomes a limit. Researchers have employed the Yin-Yang theory to discuss the characteristics and functions of vital activities. As such, we will use the Yin-Yang theory to describe how IL-17-producing Treg and IL-10-producing Th17 both hold immunosuppressive (Yin) and immunostimulant (Yang) potentials, much like how a black Yang circle exists in Yin, and vice versa, in the Yin-Yang image (Fig. 3) [221, 222].

#### The Chinese “Yin-Yang” theory and immune system

In light of the contrasting functions of immune cells and molecules, those that typically promote immune reactions are considered Yang, while those that inhibit immune reactions are considered Yin [223]. Under normal physiological conditions, the immune system maintains a delicate balance, where immunosuppressive cells and molecules like M2 macrophages, Treg, immunosuppressive cytokines such a IL-10, inhibitory receptors PD-1 and CTLA-4, and the transcription factor Foxp3, are in equilibrium with immune-activating cells and molecules, such as M1 macrophages, Th17s, and pro-inflammatory cytokines like IL-1β and IL-6, as well as immune activation-related molecules such as TLR4 and CD80, along with the transcription factor RORγT, respectively.

**Fig. 4:**
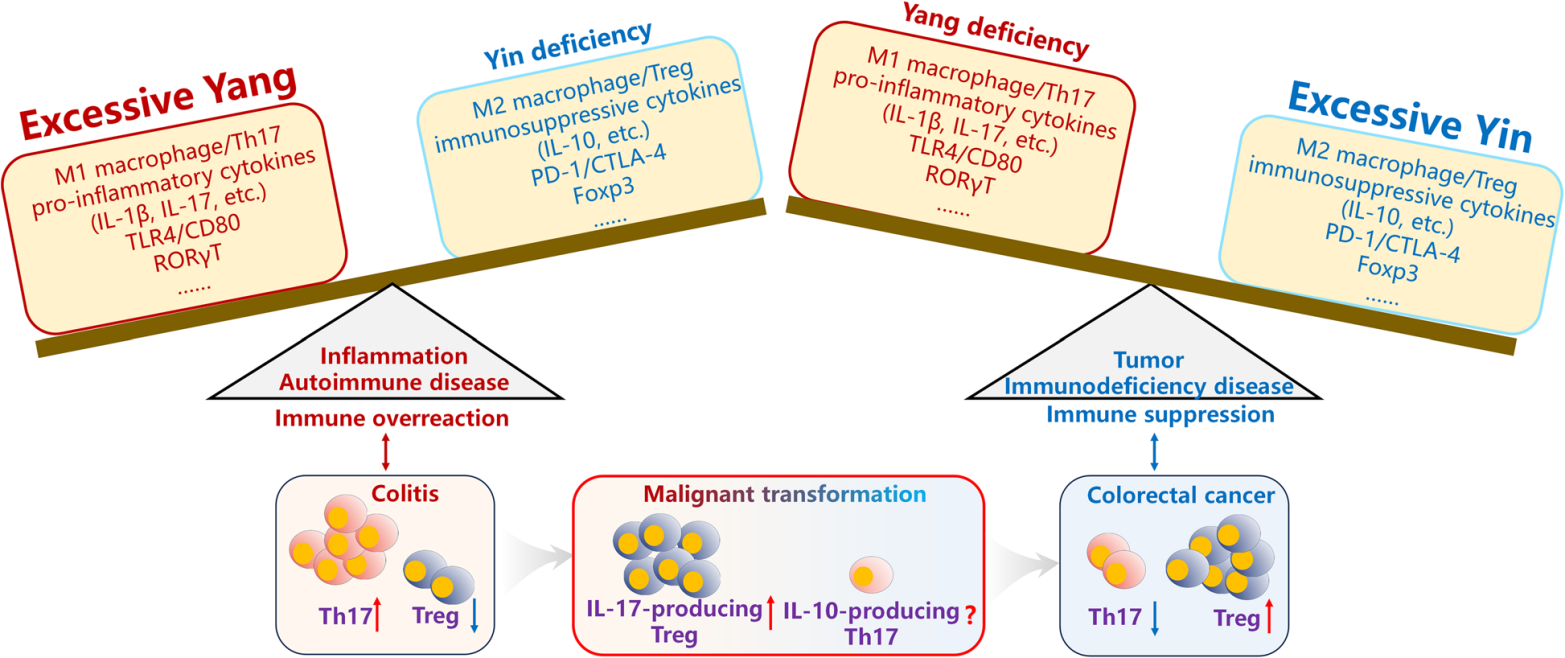
The relationship between “Yin-Yang” theory and the role IL-17-producing Treg and IL-10-producing Th17 differentiation during disease progression. Under inflammatory and autoimmune state, overexpression of immune-activation factors, coupled with a relative insufficiency of immunosuppressive factors can be signified as an excess of Yang over Yin. In tumor and immunodeficiency diseases, immunosuppressive factors surpass immune activation counterparts, indicating a dominance of Yin over Yang. During the transformation process from colitis to cancer, where there is an initial state of immune overreaction (Yang), dominated by Th17 cells during colitis, followed by a later immunosuppressive state (Yin) in colon tumors, where Tregs prevail. The yin-yang attributes of IL-17-producing Treg and IL-10-producing Th17 may offer a better understanding of the transition from immune hyperactivity to immunosuppression

However, when there is an overexpression of immune-activating cells and molecules, coupled with a relative insufficiency of immunosuppressive cells and molecules, the body may develop inflammatory and autoimmune diseases, signifying an excess of Yang over Yin [224–226]. Conversely, when immunosuppressive cells and molecules surpass immune activation counterparts, the body may be prone to tumor development and immunodeficiency diseases, indicating a dominance of Yin over Yang [227–229]. Treating various immune-related diseases by modulating immune cells to restore immunological balance aligns with the principles of traditional Chinese medicine, which aims to harmonize the Yin and Yang forces (Fig. 4).

### The association between IL-17-producing Treg and IL-10-producing Th17 and “Yin-Yang”

Current discussions regarding the relationship between Yin and Yang and the immune system revolve around their opposing attributes, drawing parallels with the mutually inhibitory functions of immune cells and molecules [230–232]. In light of the immune functions of Treg and Th17, Treg, which secrete IL-10 to inhibit immune reactions, are linked to Yin, while Th17, which secrete IL-17 to promote immune reactions, are associated with Yang. Intriguingly, as described above, IL-17-producing Treg can be seen as Yin within Yang, whereas IL-10-producing Th17 can be viewed as a manifestation of Yang within Yin. This duality of “Yin-Yang " also corresponds to their documented dual roles in promoting and inhibiting immune reactions. Furthermore, there is evidence to suggest that IL-17-producing Treg and IL-10-producing Th17 represent intermediate forms in the transformation between Treg and Th17. The discovery and examination of IL-17-producing Treg and IL-10-producing Th17 enhance the alignment between Yin-Yang theory and the immune system. In addition to their opposing characteristics, the mutual interdependence and transformation of Yin and Yang are also reflected in the study of IL-17-producing Treg and IL-10-producing Th17, deepening our understanding of the connection between Yin-Yang philosophical thought and the immune system (Fig. 3).

### IL-17-producing Treg and IL-10-producing TH17: current knowledge and unanswered questions

#### Understanding the “multiple roles” of IL-17-producing Treg and IL-10-producing Th17

Given the functional similarity of IL-17-producing Treg and IL-10-producing Th17, one may wonder if they are essentially the same cell type. Existing research indicates that they differ due to their distinct origins; IL-17-producing Treg is more likely an intermediate during the transformation of Treg to Th17 [33], while IL-10-producing Th17 arises as an intermediate during the conversion of Th17 to Tr1 [25]. Additionally, both IL-17-producing Treg and IL-10-producing Th17 participate in activating or suppressing immune responses across various physiological and pathological contexts. This seemingly contradictory outcome raises questions about the factors responsible for such dual roles. It remains to be explored whether the relative balance of Foxp3 (representing Yin) and RORγT (representing Yang) within the cells determines their distinct identities. Moreover, since IL-17-producing Treg and IL-10-producing Th17 share similar markers (IL-10^+^IL-17A^+^), it is conceivable that some of the IL-17-producing Tregs in previous studies might actually be IL-10-producing Th17 or vice versa. Various methods have been employed for detecting IL-17-producing Treg and IL-10-producing Th17, often involving modified Treg and Th17-related animal and cell models. The animal models in IL-17-producing Treg and IL-10-producing Th17 studies were summarized in Table 2. Furthermore, in vitro models including the amplification of IL-17-producing Treg [17], the induction of IL-17-producing Treg [22], IL-10-producing Th17 [27], and Tr1^exTh17^ [29] differentiation, sorting of IL-10-producing Th17 [25] have also been established by previous studies. However, there is currently no research that simultaneously detects IL-17-producing Treg and IL-10-producing Th17, necessitating further investigation to distinguish between them.

**Table 2:** In vivo models in studying IL-17-producing Treg and IL-10-producing Th17

Model	Objective	Reference
Conditional knockout mice (*Il17a*^cre^*Il10*^fl/fl^ or *Foxp3*^cre^*Rorc*^fl/fl^)	To illustrate the role of IL-17-producing Treg/IL-10-producing Th17 in disease progression	[213]
Cell transfer model using *Tcrb*^−/−^ or *Rag1*^−/−^ mice	To understand the impact of specific genes or various stimuli on IL-17-producing Treg or IL-10-producing Th17cell differentiation	[15]
CD45.1/2 mouse transplantation model	To illustrate the development process of IL-17-producing Tregs	[15, 19, 233]
Fate^+^ mice	To Sort IL-10-producing Th17, Th17, Treg, Tr1, and Tr1^exTh17^	[11, 25, 234, 235]
Th17 iFate mice	To determine whether Th17 cells differentiated into Tr1 or other types of T cells	[11, 25]
Kaede transgenic mice	To investigate the migration of intestinal IL-17-producing Tregs to parenteral tissues in response to external stimuli such as injury or stress	[15, 236, 237]

### Illustrating the detailed mechanisms involved in IL-17-producing Treg and IL-10-producing Th17 differentiation

TGF-β plays a pivotal role in inducing the differentiation of IL-17-producing Treg and IL-10-producing Th17 [12, 61], promoting both Treg and Th17 differentiation simultaneously [59]. While the mechanism of TGF-β inducing Treg differentiation is well understood, its role in further inducing IL-17-producing Treg and IL-10-producing Th17 differentiation remains to be clarified. Interestingly, from a Yin-Yang perspective, an excess of Yin may generate Yang. TGF-β-induced Treg differentiation falls within the Yin category, and TGF-β, building upon the Treg foundation, may lead to the emergence of IL-17-producing Treg, aligning with the philosophical idea of Yin and Yang transformation. Further studies are required to explain this phenomenon, and the distinct roles of various downstream Smad proteins following TGF-β induction need in-depth investigation. Additionally, various factors such as STAT, epigenetic modifications, and co-stimulatory molecules have been shown to regulate IL-17-producing Treg and IL-10-producing Th17 differentiation. Whether these pathways interact with each other and their potential relationships necessitate further exploration.

### Disease treatment by regulating the differentiation of IL-17-producing Treg and IL-10-producing Th17

The body’s immune system is in a constant state of flux, and under normal circumstances, immune cells and molecules that both activate and inhibit the immune system interact to maintain a dynamic immune balance, often described as a Yin-Yang equilibrium. Disease development disrupts this equilibrium. During the initial stages of illness, immune cells in a hyperactive state become highly activated and release pro-inflammatory factors, resulting in an excess of Yang. However, prolonged immune hyperactivity can deplete immune cells that play a role in immune promotion or prompt their transformation into immune regulatory cells, ultimately shifting the body towards an immunosuppressive state, characterized by an excess of Yin.

Consider the transformation process from colitis to cancer as an example, where there is an initial state of immune overreaction (Yang), dominated by Th17 cells during colitis, followed by a later immunosuppressive state (Yin) in colon tumors, where Treg cells prevail (Fig. 4) [238–240]. The yin-yang attributes of IL-17-producing Treg and IL-10-producing Th17 may offer a better understanding of the transition from immune hyperactivity to immunosuppression. Encouragingly, some studies have revealed the role of IL-17-producing Treg in the colitis-cancer transformation. However, whether IL-10-producing Th17 is also involved in this transition remains uncertain.

The discovery of IL-17-producing Treg and IL-10-producing Th17 provides valuable insights into the mechanisms underlying the transition process from immune activation to suppression (or vice versa) within the body. These findings offer crucial perspectives for reducing the progression of diseases like colitis to colon cancer by modulating the behavior of IL-17-producing Treg and IL-10-producing Th17.

## Data Availability

No datasets were generated or analysed during the current study.
